# Realist synthesis: illustrating the method for implementation research

**DOI:** 10.1186/1748-5908-7-33

**Published:** 2012-04-19

**Authors:** Jo Rycroft-Malone, Brendan McCormack, Alison M Hutchinson, Kara DeCorby, Tracey K Bucknall, Bridie Kent, Alyce Schultz, Erna Snelgrove-Clarke, Cheryl B Stetler, Marita Titler, Lars Wallin, Val Wilson

**Affiliations:** 1 School of Healthcare Sciences, Bangor University, Fron Heulog, Ffriddoedd Road, Bangor, UK; 2 Institute of Nursing Research/School of Nursing, University of Ulster, Shore Road, Newtownabbey, Co. Antrim, Northern Ireland; 3 School of Nursing and Midwifery, Deakin University, Burwood Highway, Melbourne, Australia; 4 Cabrini-Deakin Centre for Nursing Research, Cabrini Health, Wattletree Road, Melbourne, Australia; 5 School of Nursing, Faculty of Health Sciences, McMaster University, Hamilton, ON, Canada; 6 School of Nursing and Midwifery, Deakin University, Melbourne, Australia; 7 Eastern Health Nursing Research Unit, 5 Arnold Street, Box Hill, Melbourne, Australia; 8 EBP Concepts, Alyce A. Schultz & Associates, LLC, 5747 W Drake Court, Chandler, AZ, 85226, USA; 9 School of Nursing, Dalhousie University, 5869 University Avenue, Halifax, Canada; 10 Independent Consultant, 321 Middle Street Amherst, Massachusetts, & Health Services Department, Boston University School of Public Health Boston, Massachusetts, USA; 11 School of Nursing, University of Michigan, 400 North Ingalls Building, Ann Arbor, Michigan, MI, 48109-5482, USA; 12 Department of Neurobiology, Care Sciences and Society, Division of Nursing, Karolinska Institutet, Stockholm, Sweden; 13 Faculty of Nursing, Midwifery and Health, University of Technology Sydney, Building 10, 235-253 Jones Street, Ultimo, Australia

## Abstract

**Background:**

Realist synthesis is an increasingly popular approach to the review and synthesis of evidence, which focuses on understanding the mechanisms by which an intervention works (or not). There are few published examples of realist synthesis. This paper therefore fills a gap by describing, in detail, the process used for a realist review and synthesis to answer the question ‘what interventions and strategies are effective in enabling evidence-informed healthcare?’ The strengths and challenges of conducting realist review are also considered.

**Methods:**

The realist approach involves identifying underlying causal mechanisms and exploring how they work under what conditions. The stages of this review included: defining the scope of the review (concept mining and framework formulation); searching for and scrutinising the evidence; extracting and synthesising the evidence; and developing the narrative, including hypotheses.

**Results:**

Based on key terms and concepts related to various interventions to promote evidence-informed healthcare, we developed an outcome-focused theoretical framework. Questions were tailored for each of four theory/intervention areas within the theoretical framework and were used to guide development of a review and data extraction process. The search for literature within our first theory area, change agency, was executed and the screening procedure resulted in inclusion of 52 papers. Using the questions relevant to this theory area, data were extracted by one reviewer and validated by a second reviewer. Synthesis involved organisation of extracted data into evidence tables, theming and formulation of chains of inference, linking between the chains of inference, and hypothesis formulation. The narrative was developed around the hypotheses generated within the change agency theory area.

**Conclusions:**

Realist synthesis lends itself to the review of complex interventions because it accounts for context as well as outcomes in the process of systematically and transparently synthesising relevant literature. While realist synthesis demands flexible thinking and the ability to deal with complexity, the rewards include the potential for more pragmatic conclusions than alternative approaches to systematic reviewing. A separate publication will report the findings of the review.

## Background

The range of approaches to research review and synthesis has been growing over recent years [1]. One approach that has been growing in popularity is realist synthesis. A realist review focuses on understanding and unpacking the mechanisms by which an intervention works (or fails to work), thereby providing an explanation, as opposed to a judgment about how it works [2]. The realist approach is fundamentally concerned with theory development and refinement [2-4], accounting for context as well as outcomes in the process of systematically and transparently synthesizing relevant literature [3,4]. Given the complex, multifaceted nature of strategies and interventions used to promote evidence-informed healthcare, and the current limited understanding of their mechanisms of action, the realist approach is particularly suited to the synthesis of evidence about complex implementation interventions. Whilst the use of this method is increasing and the evidence base growing [5-8], few published studies provide a detailed account of how it has been used. This paper therefore adds to the methodological evidence base about realist synthesis by describing application of the approach of an international project team (Realist Synthesis of Implementation Strategies (ReS-IS) to synthesise evidence about knowledge translation interventions for enabling evidence-informed healthcare.

### Realist synthesis

Traditional systematic review approaches have been criticised for being too specific and inflexible [2,9,10]. This is an important consideration when examining the complexity of implementing health and social care interventions. The context of service delivery is complex, multi-faceted and dynamic, which arguably means that rarely would the same intervention work in the same way in different contexts. Consequently, conventional systematic review approaches to evaluating the evidence of whether interventions work (or not) often result in limited answers such as ‘to some extent’ and ‘sometimes’ [4,9].

Realist review has emerged as a strategy for synthesising evidence and focuses on providing explanations for why interventions may or may not work, in what contexts, how and in what circumstances [11]. For example, Greenhalgh *et al.*[6] undertook a realist review to supplement a Cochrane review of school feeding programmes. Whilst the Cochrane review provided evidence that feeding programmes work, it did not provide information about how they work and in what contexts. The findings from their realist review resulted in evidence regarding situations in which programmes may be more likely to be effective.

The realist approach is philosophically rooted in realism, which combines three social science principles: causal explanations are achievable; social reality is mainly an interpretative reality of social actors; and social actors evaluate their social reality [12]. Realism involves identifying underlying causal mechanisms and exploring how they work under what conditions [13,14]. This contextually bound approach to causality is represented as context + mechanism = outcome [15]. Therefore, it is an intuitively appealing approach to those trying to expose and unpack the complexities of contexts and interrelated mechanisms underlying implementation activity.

The aim of a realist synthesis is ‘…to articulate underlying programme theories and then to interrogate the existing evidence to find out whether and where these theories are pertinent and productive. Primary research is examined for its contribution to the developing theory…’ [4]. In the context of implementation interventions, which are usually multi-faceted and complex, when setting out to develop and implement an intervention there is always an underlying theory about how it should work: if we do X in this way, then it will bring about an improved outcome [2,4,9]. The logic underpinning the aim of uncovering underlying theories about interventions is that no deterministic theories can always explain or predict outcomes in every context [16]. Focussing on what it is about an intervention that makes it work (or not) in a given context should enable implementation researchers to work at the level of mechanisms of action. The premise is that in certain contexts individuals are likely (although not always certain) to make similar choices, and therefore particular contexts influence our choices such that reoccurring patterns emerge, *i.e.*, demi-regularities [4]. Realist review provides an approach to uncover the underlying theories that explain these demi-regularities by examining the interactions between mechanism, context, and outcome.

A realist synthesis follows similar stages to a traditional systematic review (Table 1), but with some notable differences:

1. The focus of the synthesis is derived from a negotiation between stakeholders and reviewers and therefore the extent of stakeholder involvement throughout the process is high.

2. The search and appraisal of evidence is purposive and theoretically driven with the aim of refining theory.

3. Multiple types of information and evidence can be included.

4. The process is iterative.

5. The findings from the synthesis focus on explaining to the reader why (or not) the intervention works and in what ways, to enable informed choices about further use and/or research [3].

**Table 1:** **Approach to realist review (adapted from Pawson**[9]**)**

**Stage**	**Action**	**Activity**
Define the scope of the review	Identify the question	What is the nature and content of the intervention? What are the circumstances or context of its use? What are the policy intentions or objectives? What are the nature and form of its outcomes or impacts? Undertake exploratory searches to inform discussion with review stakeholders.
	Clarify the purpose(s) of the review	Theory integrity – does the intervention work as predicted? Theory adjudication – which theories around the intervention seem to fit best? Comparison – how does the intervention work in different settings, for different groups? Reality testing – how does the policy intent of the intervention translate into practice?
	Find and articulate the programme theories	Search for relevant ‘theories’ in the literature. Draw up list of programme theories. Group, categorise or synthesise theories. Design a theoretically based evaluative framework to be ‘populated’ with evidence. Develop bespoke data extraction forms.
Search for and appraise the evidence	Search for the evidence	Decide and define purposive sampling strategy. Define search sources, terms and methods to be used (including cited reference searching). Set the thresholds for stopping searching at saturation.
	Test of relevance	Test relevance – does the research address the theory under test? Test rigour – does the research support the conclusions drawn from it by the researchers or the reviewers?
Extract and synthesise findings	Extract the results	Extract data to populate the evaluative framework with evidence.
	Synthesise findings	Compare and contrast findings from different studies. Use findings from studies to address purposes(s) of review. Seek both confirmatory and contradictory findings. Refine programme theories in the light of evidence including findings from analysis of study data.
Develop narrative		Involve commissioners/decision makers in review of findings. Disseminate review with findings, conclusions and recommendations.

The rest of this paper describes in detail the ReS-IS team’s approach to applying the realist synthesis method to a review of interventions that enable evidence-informed healthcare, including the strengths and challenges encountered in its use. A separate publication will describe the findings of the review and synthesis in detail.

## Methods

### Review purpose

Numerous interventions have been applied and tested to promote the use of research evidence in practice. Implementation of such interventions is often accompanied by complex strategies comprising support structures, resources and processes. While numerous systematic reviews have been conducted to determine the effectiveness of specific interventions [17-22], a systematic synthesis of the literature to examine the mechanisms by which such interventions work, and under what circumstances, has not been undertaken. Therefore the broad purpose of this review was to determine what interventions and strategies are effective in enabling evidence-informed healthcare. The specific purpose of the review was to establish what works, for whom, in what circumstances, and why with respect to interventions and strategies to enable evidence-informed healthcare. The purpose of the review was refined through stakeholder engagement at a knowledge utilisation colloquium meeting (http://www.uofaweb.ualberta.ca/kusp/KU0Xarchive.cfm). The stakeholders are a multi-disciplinary community of researchers, practitioners, and policy makers with expertise in knowledge translation. This community served as a stakeholder reference group that were consulted at key stages in the review process: questions formulation, tool development, and evidence synthesis, providing critique and challenge to the method and emerging findings.

## Findings

### Defining the scope of the review: Concept mining and theory formulation

This stage is fundamental to a realist synthesis because it provides the structure and framework for examining and synthesising diverse evidence [9]. The challenge of developing a framework for a realist synthesis is in finding a level of abstraction that allows reviewers to stand back from the detail and variation in the evidence, but that is also specific enough to meet the purpose of the review. For a realist synthesis, an intervention is a theory; because interventions are implemented on a hypothesis of if we do X in this way, then it will bring about an outcome. This stage involves ‘digging through’ the literature and drawing on experience to identify key terms, concepts and mid-range theories that provide some explanation about the subject of interest [4]. The resultant model must be outcome-focused because a realist synthesis is concerned with uncovering ‘what works’ within differing contextual configurations. For this review, concept mining and theory formulation was achieved through a mixture of face-to-face and virtual brain storming by the team familiar with the implementation and knowledge translation research literature.

A list of questions about interventions to promote knowledge use was developed and refined through extensive dialogue in small and larger group discussions. From these questions, the meaning of terms was clarified and concepts were identified. For example the meaning of terms such as ‘evidence-based,’ ‘knowledge,’ ‘strategy,’ and ‘intervention’ was discussed. It was agreed that an intervention was a broad concept, while strategies referred to mechanisms or approaches to achieve the intervention. Consensus was reached on the use of the term ‘evidence-informed,’ in preference to ‘evidence-based,’ on the grounds that decision making in practice, for good reasons, is not always research-based, but rather takes into account a range of factors in addition to empirical evidence [23]. We also developed a common understanding of terms related to realist synthesis (see Additional file 1).

**Figure 1:**
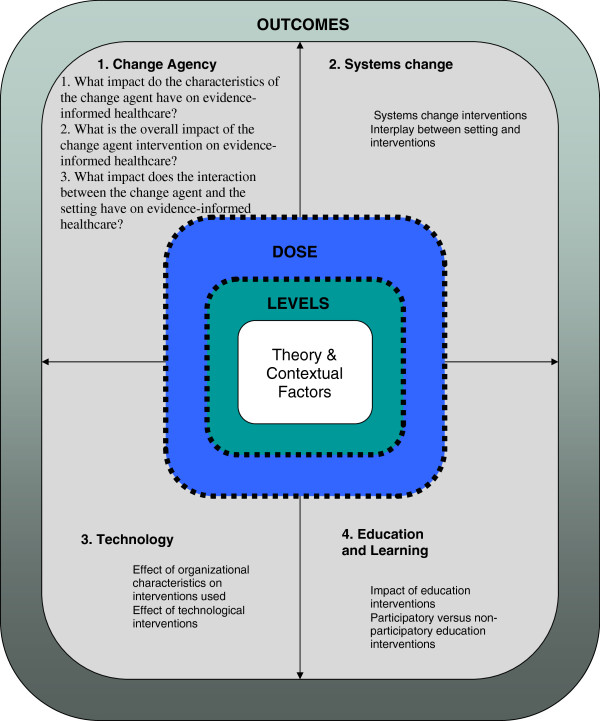
Review framework.

The group then identified common concepts among the questions developed in the previous activity. Eventually four key concepts, *i.e.*, ‘theory areas’ emerged: change agency, technology, education and learning strategies, and systems change. These concepts were then framed to construct the theoretical framework, which underwent a number of iterations before declaration of the final framework. Figure 1 presents the final framework, which includes theory and contextual factors, dose and levels as central factors because of their importance in understanding implementation interventions, and outcomes is represented as an all-encompassing factor. The term ‘dose’ was used to describe how much of an intervention would bring about change. We recognise that the term dose does not necessarily fit well within the philosophy of realism, however in the context of this review we used it to ensure we paid attention to the relative strength of action of a mechanism within a particular context. The generic questions developed earlier were then customised to each of the four theory areas and these were used to guide the review and data extraction process (see Additional file 2 for foci and questions).

The theoretical framework is made visible in a realist synthesis through the ‘data extraction forms.’ This is a unique feature of realist synthesis in that, unlike traditional systematic reviews, a bespoke set of data extraction forms are developed based on the content of the theoretical framework (see Additional file 3). The theory area questions, described previously, provided the basis for development of these forms. In order to prevent misinterpretation of the data extraction questions, guidelines for using the forms were developed to explain each section and factors to consider when applying each question during extraction of information from an article.

Pawson *et al.*[9] caution that completely comprehensive reviews may be impossible and recommend that the programme theories to be inspected be agreed upon and prioritised. As the framework reveals, the scope of our work was potentially vast. Therefore, the ReS-IS team engaged in discussion with the wider knowledge utilisation community through presentations and discussions, and as a result adopted a pragmatic approach that resulted in prioritisation of one theory area within the framework, change agency, for a first review. The programme theory questions for this theory area were:

1. What impact do the characteristics of the change agent have on evidence-informed healthcare?

2. What is the overall impact of the change agent intervention on evidence-informed healthcare?

3. What impact does the interaction between the change agent and the setting have on evidence-informed healthcare?

These questions were framed as ‘what’ questions in order to help us determine what it is about the particular actions (mechanisms) of change agents that have an impact and connect themes to action. Overall our approach to analysis was concerned with understanding why mechanisms were having an effect (or not).

### Search for, and appraisal of evidence

#### Search approach

In realist synthesis the literature needs to be scrutinised to identify studies related to the targeted ‘programme theories’ [9]. This focus does not mean that the approach to search and appraise literature is any less rigorous or systematic than approaches used in traditional reviews. The search is purposive in that for each of the theory areas the group embarked on producing a list of relevant and related search terms. The final list of terms, in conjunction with relevant indexing terms, was used to guide the searches, which were conducted by two team members in consultation with their institution’s health science librarians (see Additional file 4 for search terms and strategy).

Six online databases were searched: Medline, CINAHL, Embase, PsycInfo, Sociological Abstracts, and Web of Science. Ovid was used to execute the search within a 10-year publication period, which was considered an appropriate timeframe in the search for intervention studies. As a quality measure, one group member reviewed the indexes of 14 journals that publish articles about knowledge utilization issues. A second group member determined that relevant papers from these journals were adequately indexed in the databases selected. Additionally, using their knowledge of the literature all team members reviewed the final reference list to ensure there were no obvious omissions. Although Pawson *et al.*[9] describe the relevance of snowballing and consultation with experts as part of the realist review process, we did not have the resources to extend our search beyond the databases described above, and therefore acknowledge we may have missed some key pieces of evidence.

**Figure 2:**
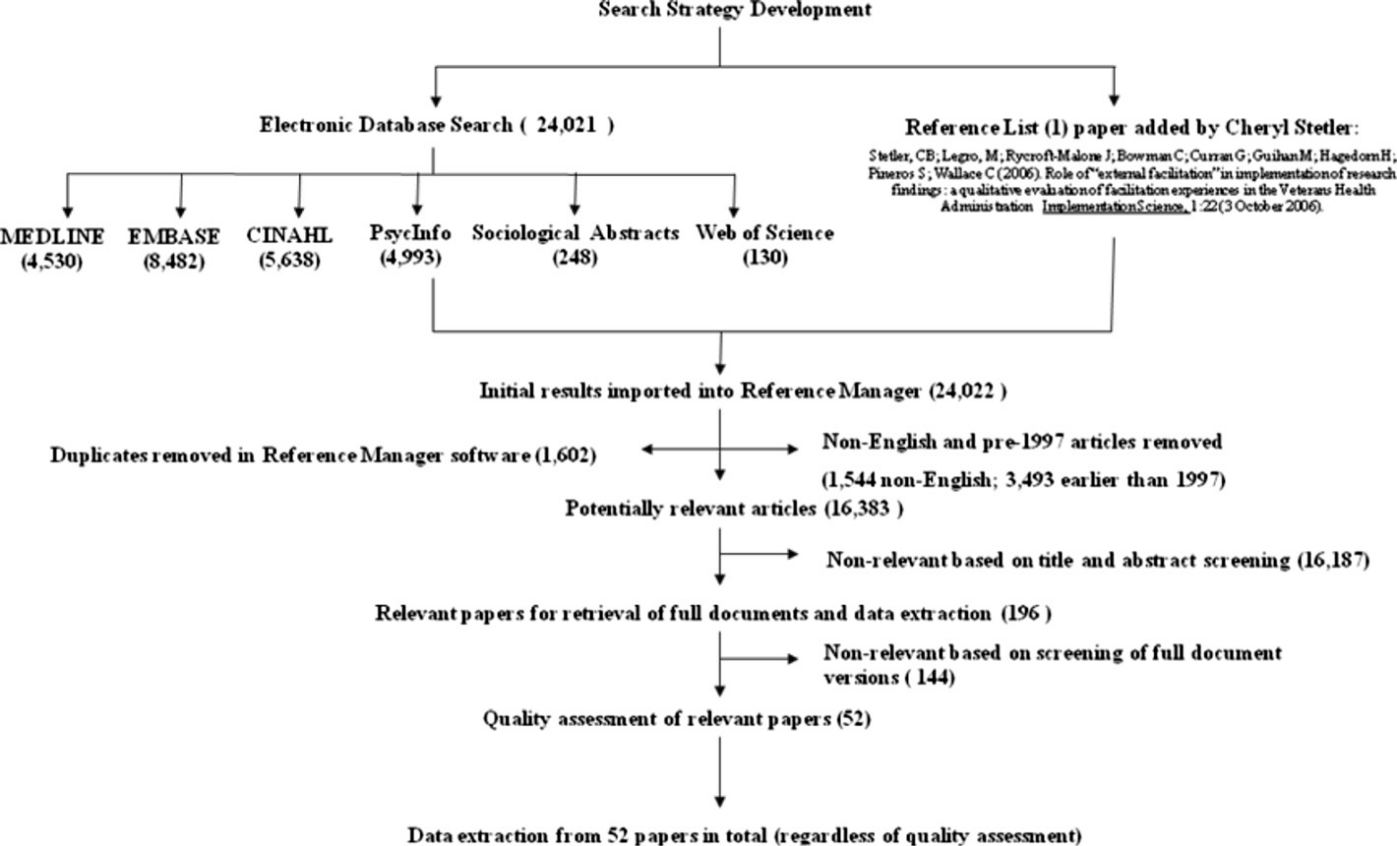
Screening process.

Search results were saved as text files and downloaded into Reference Manager Professional Version 11.0. The content of the file was then backed-up to a secure server. Over 24,000 electronic references were returned from the change agents search strategies, which were screened through a process summarised in Figure 2.

The preliminary screen was intentionally inclusive to capture all articles potentially relevant to the review purpose, including those outside of healthcare. We erred on the side of inclusion wherever a title appeared to be even potentially relevant to the change agent/agency concept or any of the terms of reference developed in relation to the change agency component of the theoretical framework. At this stage, all seemingly relevant papers were retrieved in full-text for a more detailed relevance test: ‘Is the evidence provided in this theory area good and relevant enough to be included in the synthesis.’

Using criteria for exclusion, in the next stage we undertook a preliminary screening of the article titles retrieved in the search and reduced the list of potentially relevant papers to 196. Second-level screening resulted in the exclusion of a further 144 papers, bringing the final number of papers relevant to this theory area to 52. This process was managed by two team members, in consultation with the wider group as appropriate. Our consistent reference point was the review framework and related theory area questions. Therefore decisions about what was eventually included and excluded was managed through discussions about whether the papers were of direct relevance to interventions that had been evaluated within a healthcare context.

The primary reasons for exclusion of papers at this level included:

1. They were not research based (using the broadest definition of research, *i.e.*, demonstrating a systematic approach to inquiry).

2. They were purely an anecdotal account.

3. They were not relevant to healthcare, *e.g.*, were based on an unrelated field such as banking (we did not have resources to undertake a review of the wider literature).

#### Appraisal

To test the usability and functionality of the data extraction form and to promote a consistent approach to data extraction, the tool was pre-tested, by all group members, on two purposefully selected articles. Pre-testing resulted in some minor modifications to the instrument prior to commencement of the review process. The group divided into five subgroups and the references related to the change agent theory area were divided evenly across the subgroups for appraisal.

In contrast to other review processes, in a pure realist synthesis no literature is excluded (unless it does not relate to any of the theory areas). Because a paper is excluded in one theory area does not mean that it is necessarily excluded in other theory areas. Therefore, exclusion criteria at this level need to be explicit and a clear rationale needs to be documented for each article that is excluded. Consistent with Pawson’s suggestions, in this review the test for inclusion was: Is the evidence provided in this theory area ‘good and relevant enough’ to be included (consider issues of sample size, data collection, data analysis, and claims made)?

Discrepancies in opinions about the relevance of articles were resolved through discussion amongst the review group. Questions about relevance were guided by the theory area questions, and how particular pieces of evidence did or did not inform these. Final decisions erred on inclusiveness, rather than exclusion.

### Extraction and synthesis

#### Data extraction

If considered relevant, data were extracted from the article and then peer reviewed and checked by a second member of the subgroup. Even though the initial review wave comprised articles related to the change agent theory area, questions relating to each theory were applied during the extraction process because many of the articles reported aspects that were also relevant to the other theory areas. As the contents of the review’s theoretical framework were embedded in data extraction forms, these provided a template to ‘interrogate’ the papers. When extracting data, if an article did not include information relevant to a question in the form, the extractor recorded ‘Not reported.’ Direct quotations from the article were often most informative and were accompanied by the page number from which the quote was taken. Data pertaining to the target population and discipline for each study were also extracted.

The aim of the data extraction process is to populate the evaluative framework with evidence. Therefore, once all subgroups had completed data extraction for the articles deemed relevant to the review purpose, the content from each group’s data extraction tables was amalgamated to form a single data extraction table including all articles addressing change agency.

#### Data synthesis

The basic task of the synthesis process is to refine the programme theory; *i.e.*, to determine what works, for whom, in what circumstances, in what respects and why. Pawson *et al.*’ publications provide little guidance on how to approach data synthesis. They suggest that synthesis should focus on four dimensions: questioning the integrity of a theory, adjudicating between competing theories, considering the same theory in comparative settings, or comparing the ‘official’ theory with actual practice [4,9].

In this review we developed an approach to synthesis based on the principles of realist evaluation [15], including the following steps:

1. Organisation of extracted data into evidence tables,

2. Theming by individual reviewers,

3. Comparison of reviewers’ themes for a specific article and formulation of chains of inference from the identified themes,

4. Linking of the chains of inference, and tracking and linking of articles

5. Hypothesis formulation.

This process is summarised in Additional file 5.

At a face-to-face meeting, sub-groups undertook a process of analysis, focussed on addressing the three programme theory questions (see Figure 1 and ‘Defining The Scope’ section above).

Each question was assigned to a subgroup, and its members’ independently themed relevant data extracted from each article. The subgroup then collated the themes identified by each of the members. At this point, emerging findings were challenged, and contrary examples sought. From here the subgroup members identified chains of inference. A chain of inference is a connection that can be made across articles based on the themes identified. Over a period of time, through virtual and face-to-face meetings, chains of interference were developed and refined. This process was both inductive and deductive. We did not get to the stage of retroduction within this review, which could therefore be viewed as a limitation of the review process reported here. Further work would need to be undertaken on the hypotheses to make better inferences about generative mechanisms.

The group then formulated hypotheses regarding the chains of inference. Thus, themes were linked to chains of inference, which were then linked to a hypothesis. Further, all papers were explicitly linked to chains of inference and hypotheses. Additional file 5 includes full details of the stages of synthesis, and Additional file 6 presents full details of the hypotheses linked to themes, chains of inference, and papers. Tables 2 and 3 provide a summary of these stages.

**Table 2:** Chains of inference linked to themes and original articles

**Chains of Inference**	**Derived from the following themes**	**Articles**
**Knowledge**	Professional qualifications Expert knowledge Knowledgeable Local knowledge Research Knowledge Practice knowledge	1, 3, 6, 7, 10, 11, 13, 14, 15, 16, 18, 19, 20, 21, 22, 23, 25, 29, 35, 36, 37, 39
**Skills**	Communication skills Leadership skills Thinking skills Clinical skills Cognitive skills Evaluation skills Political skills Facilitation Skills Reflective skills	2, 4, 5, 6, 7, 8, 9, 10, 11, 12, 13, 14,15,16, 17, 18, 19, 20, 21, 22, 24, 25, 27, 28, 32, 33, 34, 36, 38, 39, 40
**Personal Characteristics**	Role model Positive attitude Responsibility/accountability Respected Information Seeking Positive Attitude Accessible Age Teacher Culturally compatible Objectivity Years of experience	1, 2, 4, 6, 7, 8, 13, 14, 15, 16, 17, 18, 22, 28, 29, 30, 31, 32, 33, 35, 36, 37, 38, 39
**Social Interaction**	Social Influence Networking Shared Ownership	5, 8, 12, 15, 18, 31, 39, 40,

**Table 3:** Hypotheses linked to chains of inference

**Hypotheses**	**Chain of Inference (theory level)**	**Chain of inference (sub-theory level)**	**Themes from the literature**	**Papers addressing the theme**
An opinion leader and his/her personal characteristics are dependent on contextual factors in order to have an impact on E-IHC.A facilitator and his/her personal characteristics are dependent on contextual factors in order to have an impact on E-IHC.	The nature of the relationship between the change agent’s personal characteristics, the role adopted, and contextual influences and the impact of E-IHC.	Roles Personal Characteristics Contextual Factors	Opinion Leader (OL) Facilitator (FAC) Change agent (CA)	*Papers with mixed and positive effects, only: *6 OL (Wright, Chaillet, Curran, Moore, Davies, Majumdar) Six FAC (internal/external and external facilitators included), (Stetler, Cranney, Gerrish, Milner, Thomas, Hutt) Total 18 CA papers, 12 OL and FAC

#### Development of narrative

Typically the writing of the review follows the theoretical framework model developed. For this review, the narrative was organised according to hypotheses generated within the Change Agency theory area, with the data for each theme linked in two ways; to one another within each hypothesis, and also across hypotheses. Whilst the findings from this theory area will be presented in a separate publication, the following presents a summary to illustrate our approach to analysis and synthesis.

##### Step one

Data from extraction tables were summarised and organised into theory area and questions related tables, for example, in relation to the question ‘what impact do the characteristics of the change agents have on knowledge utilization,’ the table would include extracts of data, and the link back to the source papers.

##### Step two

These data were then themed, those themes were challenged and contrary evidence sought. In relation to the characteristics of change agents a number of emerging issues emerged that could be considered as relevant conditions for change agency within an opinion leader role for example, such as confidence, years of experience, level of qualification (educated to post-graduate level), and willingness to work collaboratively.

##### Step three

Looking for chains of inference (connections) across extracted data and themes. Through an iterative process, connections were looked for across data/themes to build up a cumulative picture. For example, were the opinion leader conditions evident in papers about other change agent roles—this resulted in a larger list factors, including, for example attitude, expert knowledge, gender, age, and cultural compatibility (see Table 2 for more examples), leadership, being embedded, tailoring, partnerships, influence, culture, support, and resources.

##### Step four

Hypotheses formation (mechanism, context, outcome chains). The output from steps three and four resulted in a cumulative picture of potential mechanisms, contexts and outcome chains (hypotheses), which could be linked back to source evidence (see Table 3 for an example). Our review indicates that, for example, change agents who are adequately supported and resourced (context) who role model the practices they espouse (mechanism) may impact more positively on achieving evidence-informed healthcare (outcome).

The hypotheses acted as synthesised statements of findings against which the previous stages of analysis could be presented. The narrative was developed around each hypotheses, and summarised the nature of the context, mechanism and outcome links, and the characteristics of the evidence underpinning them

Pawson *et al*. [9] do not suggest that ‘recommendations’ *per se* are developed from realist synthesis, as the purpose is not to determine ‘best’ practice, but to describe the relationships between interventions and the contexts in which those interventions occur. They do however suggest that stakeholders are engaged both in the process of ‘validating’ the emerging findings and in dissemination activities. To that end, our review approach and the developing findings were shared with a community of knowledge utilisation researchers and practitioners at an annual colloquium in 2009. This process helped to refine the focus and presentation of the narrative from the programme theory area. It also validated our view that some new insights about change agency had emerged from this review process, which will be reported in a separate paper.

## Discussion

There are few published examples of realist syntheses and those that exist do not include a detailed account of the approach used because authors tend to focus on the dissemination of findings within publications [6,7,24-27]. This lack of information about application of the realist approach is unhelpful to a novice realist reviewer. To fill this gap, this paper presents in some detail the approach we took to conduct a realist synthesis of evidence about the effect of change agency on evidence-informed healthcare. Change agency is a complex implementation intervention, which made realist synthesis an appropriate approach for unpacking its effects within different contexts and groups. However, undertaking this review was not without its challenges, not least because of the practicalities of working as an unfunded and geographically dispersed group.

Our approach deviated in some respects from the approach recommended by Pawson *et al.* For example, Pawson *et al*. do not advocate a comprehensive literature search, or double reviews and data extraction. In this sense we developed a hybrid approach that was fundamentally rooted in realist synthesis philosophy and principles (*i.e.*, theory led, purposive, iterative, stakeholder involvement), but which also drew on some of the practices of traditional systematic reviewing.

One of the strengths of realist review is the approach’s firm roots in philosophy and social sciences [2,9]. Rather than being a method or formula, it is a ‘logic of enquiry’ [9], which enables a flexible, all-embracing approach to explanation of what works for whom in what circumstances and in what respects. Rather than controlling for real life events, realist synthesis provides a framework for working with and untangling the complexity of real-life implementation. This allows for an equal focus on what works, as much as what does not work, in an attempt to learn from failures and maximise learning across policy, disciplinary and organisational boundaries. Furthermore, realist synthesis is inherently stakeholder driven, which facilitates engagement and the inclusion of multiple perspectives.

The strengths of realist synthesis underpin its limitations. Realist synthesis is premised on a set of principles rather than a formula, and whilst this allows for flexibility and inclusivity, it means that the findings from a review are theoretically transferable. For example, it follows that if the appraisal and data extraction needs to be bespoke to the particular review questions that arise from the theoretical framework, these will be different for each review. Furthermore, given that the fundamental interest in realist synthesis is about finding out what works in what contexts, the recommendations one can make will not be generalisable. Rather a realist review results in findings that are theoretically transferable; ideas (‘theories’) that can be tested in different contexts, with different stakeholders.

Pawson *et al*. [2] suggest that realist syntheses are not for novices. Unlike a Cochrane review, for example, which relies on standardised protocols and tools, the demands on a realist synthesiser are different. For example, quality assurance within realist synthesis is dependent on reviewers’ explicitness and reflexivity. During this review, we kept a log of the process and decisions made throughout the process, which we developed into a technical report. In addition, we undertook a more formal reflective process during the review because members of the group had varying experiences of realist review. This involved reflecting on questions about what was going well, what was going less well, as well as engaging in group learning activities. Throughout the review process, we had large and small group discussions that provided the opportunity for building in checks and balances, and for explicating processes. In turn, this requires a high level of expertise in reasoning, research methods and quality appraisal, and expertise in the subject area. The complexity of the realist synthesis approach means that it is time-consuming and human resource intensive, and for those reasons a potentially expensive endeavour.

## Conclusions

Realist synthesis is a new but emerging approach to evidence review. In this paper, we have described our use and development of the approach. It is particularly appropriate for unpacking the impact of complex interventions because it works on the premise that one needs to understand how interventions work in different contexts, and why. It is not an easy option. Realist review demands much of the reviewer, including an ability to think flexibly and deal with complexity. There is not one prescribed approach to doing a realist synthesis; rather, there is a set of principles that the reviewer must particularise to the issue being explored whilst being sympathetic to the philosophy of realism. This presents unique challenges, but with it, the opportunity to develop more pragmatically insightful conclusions than those produced by some other approaches to systematic reviewing.

## Competing interests

Bridie Kent is an Associate Editor for Implementation Science; all decisions on this manuscript were made by other editors.

## Authors’ contributions

BM and JRM led the project. All authors participated in defining the scope of the review. KD and ESC executed the search. The appraisal of evidence and data extraction were undertaken by all authors. All authors were involved in the analysis process. AMH led the documentation of the study process. KD led the development of the narrative. JRM wrote the first draft of the paper; BM, AH, and KD commented on it. All authors provided feedback on various drafts, and read and approved the final manuscript.

## Supplementary Material

Additional file 1 Terms related to realist synthesis. Click here for file

Additional file 2 Review foci and questions. Click here for file

Additional file 3 Data extraction form. Click here for file

Additional file 4 Search terms and strategy. Click here for file

Additional file 5 Stages of synthesis. Click here for file

Additional file 6 Hypotheses linked to themes, chains of inference and papers. Click here for file
