# Supplementary material for: Realist synthesis: illustrating the method for implementation research
Source: Implement Sci. 2012 Apr 19;7:33. doi: 10.1186/1748-5908-7-33 (PMC3514310; doi:10.1186/1748-5908-7-33)
Supplement: Additional file 1 — Terms related to realist synthesis. [file 1748-5908-7-33-S1.doc]

**Terms and Definitions Used in Realist Synthesis**

***Purpose of realist synthesis****:*

‘…to articulate underlying programme theories and then to interrogate the existing evidence to find out whether and where these theories are pertinent and productive. Primary research is examined for its contribution to the developing theory…’ (p. 74, 2006)

**Theory** – different from the traditional approach to defining theory – for realist synthesis an intervention is a theory – because they are always based on an hypothesis – if we do X in this way, then it will bring about an improved outcome. If we use a hand held computer to deliver a just in time message to nurses to prompt them to deliver a particular type of care for patients with CHD, it will result in better patient outcomes. What realist synthesis would be interested in is what is about the hand held computer just in time message, that works, with whom (nurses? doctors? patients? Policy makers?) and in what circumstances (a home?, in the car on the way to the patient?, in the hospital?). Realist synthesis then is about refining and developing theory in a particular way (in a more grounded theory type of way).

**Methods** (as proposed by Pawson, 2006) – some key issues

- 1. Searches are ‘more intricate’ (than standard systematic review processes) because they are linked to the questions and sub-questions. The aim of the search is to initialise theory building.
  2. Pragmatic approach to searching the literature – scan the literature as thoroughly as possible – but also need to draw the line somewhere – takes a purposive sampling logic to retrieve materials purposively to answer specific questions or test particular theories.
  3. Purposive sampling achieves closure through ‘theoretical saturation’ – cease when sufficient evidence has been assembled to satisfy the theoretical need or to answer the question.
  4. Pawson doesn’t necessarily advocate the use of a quality appraisal check list – uses a guiding principle – the appraisal criteria should be subordinate to the usage to which the study is put (Pawson 2006, p. 87) i.e. appraise studies according to the contribution that each one makes to the developing synthesis. Quality assessment should be review specific and theory driven. The reviewer should ask – ‘is this study good enough to provide some evidence that will contribute to the synthesis’ (p. 88).
  5. Make an assessment of relevance - is the content appropriate to add to the review?
  6. Make an assessment of rigour – is it of sufficient quality to help in clarifying a particular question/theory? Pawson suggests that even in studies that are ‘technically deficient’ there may still be some trustworthy nuggets of information that contribute to the overall synthesis.
  7. No equivalent to data matrix (re trad review) – a more elongated model of extraction because 1) the original sources are used for different purposes, and 2) each source is expected to contribute different information to the synthesis (p. 91) -erecting an explanation rather than loading different bits of information onto a conveyer belt.
  8. Suggests annotating papers – give passages an appropriate label. (p. 92-93)
  9. Synthesising – the task is to refine theory – through testing, adjudicating and comparing (p. 97-99)
