# Supplementary material for: Realist synthesis: illustrating the method for implementation research
Source: Implement Sci. 2012 Apr 19;7:33. doi: 10.1186/1748-5908-7-33 (PMC3514310; doi:10.1186/1748-5908-7-33)
Supplement: Additional file 2 — Review foci and questions. [file 1748-5908-7-33-S2.doc]

**Theory areas**

The ReS-IS model has four theoretical areas and 13 theoretical foci.

*Theory area 1 - Properties of change agency in evidence-informed health care (E-IHC)*

- What impact do the characteristics of the change agent have on E-IHC
- What is the overall impact of the change agent intervention on E-IHC
- What impact does the interaction between the change agent and the setting have on E-IHC

*Theory area 2 –system change in E-IHC*

- What impact do characteristics of the systems change intervention(s) have on E-IHC?
- What is the overall impact of the system change intervention(s) used?
- What impact does the interaction between the system change and the setting have on E-IHC?
- What impact do senior leadership roles have in creating practice environments that integrate daily use of evidence at the point of care delivery?

*Theory area 3 – properties of technologies (paper & electronic) used in E-IHC*

- What impact do the characteristics of the technological intervention(s) have on E-IHC?
- What is the overall impact of the technological intervention(s) used?
- What impact does the interaction between the technological intervention and the setting have on E-IHC?

*Theory area 4 – education interventions in E-IHC*

- What impact do the characteristics of the education intervention(s) have in enabling E-IHC?
- What is the overall impact of the education intervention(s) used?
- What impact does the interaction between the education intervention and the setting have on E-IHC?
