# Supplementary material for: Realist synthesis: illustrating the method for implementation research
Source: Implement Sci. 2012 Apr 19;7:33. doi: 10.1186/1748-5908-7-33 (PMC3514310; doi:10.1186/1748-5908-7-33)
Supplement: Additional file 3 — Data extraction form. [file 1748-5908-7-33-S3.doc]

**Data Extraction Form used for Theory Area 1**

## ReS-IS Working Group data extraction form

| Full reference: |
| --- |
| Theory area 1 – Properties of change agent in evidence-informed healthcare |
| What are the characteristics of the change agent? |
|  |
| What impact do the characteristics of the change agent have on evidence-informed healthcare? |
|  |
| What is the change agent intervention? |
|  |
| What is the overall impact of the change agent intervention on evidence-informed healthcare? |
|  |
| What is the interaction between the change agent and the setting? |
|  |
| What impact does the interaction between the change agent and the setting have on evidence-informed healthcare? |
|  |
| Is the evidence provided in this theory area good and relevant enough to be included in the synthesis (consider issues of sample size, data collection, data analysis and claims made) |
|  |

| Theory area 2 –system change in evidence-informed healthcare |
| --- |
| What are the characteristics of the systems change intervention(s)? |
|  |
| What impact do characteristics of the systems change intervention(s) have on evidence-informed healthcare? |
|  |
| What is the system change intervention(s) used? |
|  |
| What is the overall impact on KU of the system change intervention(s) used? |
|  |
| What is the interaction between the system change and the setting? |
|  |
| What impact does the interaction between the system change and the setting have on evidence-informed healthcare? |
|  |
| What impact do senior leadership roles have in creating practice environments that integrate daily use of evidence at the point of care delivery? |
|  |
| Is the evidence provided in this theory area good and RELEVANT ENOUGH to be included in the synthesis (consider issues of sample size, data collection, data analysis and claims made) |
|  |

| Theory area 3 – properties of technologies (paper & electronic) used in evidence-informed healthcare (technology = mechanisms not mediated through an INDIVIDUAL PERSON) |
| --- |
| What are the characteristics of the technological intervention(s)? |
|  |
| What impact do the characteristics of the technological intervention(s) have on evidence-informed healthcare? |
|  |
| What is the technological intervention(s) used? |
|  |
| What is the overall impact of the technological intervention(s) used? |
|  |
| What is the interaction between the technological intervention and the setting? |
|  |
| What impact does the interaction between the technological intervention and the setting have on KU? |
|  |
| Is the evidence provided in this theory area good and relevant enough to be included in the synthesis (consider issues of sample size, data collection, data analysis and claims made) |
|  |

| Theory area 4 – education interventions in evidence-informed healthcare |
| --- |
| What impact do the characteristics of the education intervention(s) have in enabling evidence-informed healthcare? |
|  |
| What are the characteristics of the education intervention(s)? |
|  |
| What is the education intervention(s) used? |
|  |
| What is the overall impact of the education intervention(s) used? |
|  |
| What is the interaction between the education intervention and the setting? |
|  |
| What impact does the interaction between the education intervention and the setting have on KU? |
|  |
| Is the evidence provided in this theory area good and relevant enough to be included in the synthesis (consider issues of sample size, data collection, data analysis and claims made) |
|  |

| Is there evidence of particular theoretical perspective(s) impacting on the effectiveness of the intervention? |
| --- |
|  |
| Is there evidence of contextual factors impacting on the effectiveness of the intervention? |
|  |
| Is there evidence of the level of the intervention impacting on the effectiveness of the intervention? |
|  |
| Is there evidence of the intervention dose impacting on the effectiveness of the intervention? |
|  |
