# Supplementary material for: Realist synthesis: illustrating the method for implementation research
Source: Implement Sci. 2012 Apr 19;7:33. doi: 10.1186/1748-5908-7-33 (PMC3514310; doi:10.1186/1748-5908-7-33)
Supplement: Additional file 4 — Search terms and strategy. [file 1748-5908-7-33-S4.doc]

*Change Agents*

The characteristics (attributes, types, roles) of change agents that effect successful change in KU. The individual (characteristics, attributes, types, quantities) that use strategies (type, dose) effecting successful change in **organisational** KU and **personal** KU.

Terms used interchangeably to refer to change agents:

Opinion leader

Facilitator/ion

Education outreach worker

Academic detailer

Practice developer

Clinical Educator

Change agent

Knowledge broker

Champion

Innovator

Boundary spanner

Advocate

Expert

Transformational leader

Consultant

Coach

Educator

Nurse researcher

EBP champion

Staff developer

Professional practice developer

*Systems Change*

Characteristics of an organisation (system, type, senior leadership) that is receptive to implementing (interventions, strategies) and sustaining the use of evidence in practice.

Related Terms:

Leadership (transformational)

Culture

Context

Climate

Magnet

Organisational readiness

Receptiveness

Learning organisations

Resources

Role clarity

Team work

Rewards/recognition/incentives

Organisational structures

Social networks

Collaboration

Multidisciplinary

Interdisciplinary

Decision making processes

Organisational structure

Organisational innovativeness

Organisational complexity

Soft systems

Hard systems

Boundaries

Locality

*Technology*

The technologies, including paper and electronic based technology (type, dose), used to enable and foster evidence informed healthcare (EIH).

Related Terms:

Audit and feedback

Guidelines

Protocols

Standards

Policies

Procedures

Reminders

Care pathways/clinical pathways/critical pathways/care maps/integrated pathways

Systematic reviews

Decision-support

Algorithms

Hand held technology

Web-based resources

Innovation

*Education & Learning*

Terms:

Reflective learning

Didactic

Pedagogic

Pedagogical

Pedagogy

Education

Training

Problem based learning

Action learning (sets)

Academic detailing

Professional development

Practice development

Adult learning

Reflection

Androgogy

Androgogical

Work-based-learning

Experiential

*Levels* – individual, organisational, systems-wide

*Theory* - models and frameworks that support change interplay within and between the individual, organisational and systems

*Context* – the organisational factors that impact on the use of EIH…type, support, moral, staffing mix, etc.

Defined terms used in realistic synthesis

- views
- values
- beliefs
- decision making
- individual
- successful
- unsuccessful
- setting
- context
- change agent (individual/group)
- implementation strategies
- amount (dose, frequency, intensity) of a strategy/technology
- individual mastery
- organisational mastery
- outcomes
- technological intervention
- communication technology
- organisation
- receptive organisation
- organisational readiness
- discipline
- role – front line care, middle management, senior leadership….
- education and learning strategies
- participatory strategies
- non-participatory strategies
- evidence informed health care
- knowledge utilisation
- knowledge translation
- knowledge transfer and exchange
- research utilisation
- theory contextual factors

Levels

1. What impact do the views, values & beliefs of different stakeholders have on the intervention??
2. What impact do decision making characteristics of individuals have on an intervention
3. What impact do decision making characteristics of teams have on an intervention?
4. What impact do decision making characteristics of organisations have on an intervention?
5. What impact do decision making characteristics of regulatory agencies have on an intervention?
6. What impact do the characteristics of different professional groups have on the perceived usefulness of an intervention?
7. What interventions are effective in increasing receptivity/sensitivity with the target of the intervention?

So, based on this – levels are: different stakeholders, individuals, teams, organisations, regulatory organisations, professional groups

**Appendix 1**

**Search Strategies**

Search Strategies for Medline, Embase, CINAHL, PsycInfo, Sociological Abstracts, Web of Science

Executed March 7 – March 9, 2007

Database: Ovid MEDLINE(R)

Search Strategy:

--------------------------------------------------------------------------------

1 opinion leader.mp. [mp=title, original title, abstract, name of substance word, subject heading word] (44)

2 facilitat$.mp. [mp=title, original title, abstract, name of substance word, subject heading word] (147970)

3 education outreach$.mp. [mp=title, original title, abstract, name of substance word, subject heading word] (30)

4 academic detail$.mp. [mp=title, original title, abstract, name of substance word, subject heading word] (154)

5 practice develop$.mp. [mp=title, original title, abstract, name of substance word, subject heading word] (439)

6 clinical educator.mp. [mp=title, original title, abstract, name of substance word, subject heading word] (27)

7 change agen$.mp. [mp=title, original title, abstract, name of substance word, subject heading word] (314)

8 knowledge broker$.mp. [mp=title, original title, abstract, name of substance word, subject heading word] (9)

9 champion.mp. [mp=title, original title, abstract, name of substance word, subject heading word] (438)

10 innovator.mp. [mp=title, original title, abstract, name of substance word, subject heading word] (245)

11 boundary spann$.mp. [mp=title, original title, abstract, name of substance word, subject heading word] (25)

12 advoca$.mp. [mp=title, original title, abstract, name of substance word, subject heading word] (43602)

13 expert.mp. [mp=title, original title, abstract, name of substance word, subject heading word] (24830)

14 transformational leader$.mp. [mp=title, original title, abstract, name of substance word, subject heading word] (155)

15 consultan$.mp. [mp=title, original title, abstract, name of substance word, subject heading word] (12050)

16 exp *Consultants/ (2344)

17 coach$.mp. [mp=title, original title, abstract, name of substance word, subject heading word] (2663)

18 educator.mp. [mp=title, original title, abstract, name of substance word, subject heading word] (1755)

19 exp *Health Educators/ (39)

20 1 or 2 or 3 or 4 or 5 or 6 or 7 or 8 or 9 or 10 or 11 or 12 or 13 or 14 or 15 or 16 or 17 or 18 or 19 (230837)

21 knowledge transfer.mp. [mp=title, original title, abstract, name of substance word, subject heading word] (140)

22 knowledge translation.mp. [mp=title, original title, abstract, name of substance word, subject heading word] (63)

23 knowledge uptake.mp. [mp=title, original title, abstract, name of substance word, subject heading word] (11)

24 knowledge utilization.mp. [mp=title, original title, abstract, name of substance word, subject heading word] (38)

25 knowledge exchange.mp. [mp=title, original title, abstract, name of substance word, subject heading word] (36)

26 research uptake.mp. [mp=title, original title, abstract, name of substance word, subject heading word] (8)

27 research utilization.mp. [mp=title, original title, abstract, name of substance word, subject heading word] (346)

28 knowledge utilisation.mp. [mp=title, original title, abstract, name of substance word, subject heading word] (3)

29 research utilisation.mp. [mp=title, original title, abstract, name of substance word, subject heading word] (21)

30 evidence based$.mp. [mp=title, original title, abstract, name of substance word, subject heading word] (31693)

31 evidence informed$.mp. [mp=title, original title, abstract, name of substance word, subject heading word] (29)

32 innovation.mp. [mp=title, original title, abstract, name of substance word, subject heading word] (25045)

33 research use$.mp. [mp=title, original title, abstract, name of substance word, subject heading word] (615)

34 research translation.mp. [mp=title, original title, abstract, name of substance word, subject heading word] (8)

35 research trans$.mp. [mp=title, original title, abstract, name of substance word, subject heading word] (79)

36 exp *Organizational Innovation/ or exp *"Diffusion of Innovation"/ (6981)

37 21 or 22 or 23 or 24 or 25 or 26 or 27 or 28 or 29 or 30 or 31 or 32 or 33 or 34 or 35 or 36 (57602)

38 20 and 37 (4530)

39 from 38 keep 1-1000 (1000)

***************************

Database: EMBASE

Search Strategy:

--------------------------------------------------------------------------------

1 opinion leader.mp. (42)

2 facilitat$.mp. (135598)

3 education outreach$.mp. (23)

4 exp *medical education/ (34690)

5 academic detail$.mp. (155)

6 practice develop$.mp. (163)

7 exp *Clinical Education/ or clinical educator.mp. (2010)

8 change agen$.mp. (158)

9 knowledge broker$.mp. (6)

10 champion.mp. (259)

11 innovat$.mp. (20668)

12 boundary spann$.mp. [mp=title, abstract, subject headings, heading word, drug trade name, original title, device manufacturer, drug manufacturer name] (17)

13 advoca$.mp. [mp=title, abstract, subject headings, heading word, drug trade name, original title, device manufacturer, drug manufacturer name] (19519)

14 expert.mp. or *EXPERT SYSTEM/ or *MEDICAL EXPERT/ or *EXPERT NURSE/ (21317)

15 *Leadership/ or transformational leader$.mp. (1010)

16 consultan$.mp. [mp=title, abstract, subject headings, heading word, drug trade name, original title, device manufacturer, drug manufacturer name] (7935)

17 coach$.mp. (2234)

18 *HEALTH EDUCATOR/ or *DIABETES EDUCATOR/ or educator.mp. (942)

19 1 or 2 or 3 or 4 or 5 or 6 or 7 or 8 or 9 or 10 or 11 or 12 or 13 or 14 or 15 or 16 or 17 or 18 (237986)

20 knowledge trans$.mp. (197)

21 knowledge transfer.mp. (115)

22 knowledge translation.mp. (47)

23 knowledge uptake.mp. (8)

24 knowledge utilization.mp. (13)

25 knowledge exchange.mp. (19)

26 research uptake.mp. (4)

27 research utilization.mp. (55)

28 research utilisation.mp. (11)

29 knowledge utilisation.mp. (3)

30 evidence-based$.mp. or exp *Evidence Based Practice/ (40261)

31 exp *EVIDENCE BASED NURSING/ or exp *EVIDENCE BASED PRACTICE CENTER/ or exp *EVIDENCE BASED MEDICINE/ (7320)

32 innovation.mp. (4209)

33 exp *NURSING EVALUATION RESEARCH/ or exp *MEDICAL RESEARCH/ or exp *RESEARCH UTILIZATION GROUP/ or exp *NURSING RESEARCH/ or exp *RESEARCH/ (39951)

34 research trans$.mp. (62)

35 organizational innovation.mp. (21)

36 20 or 21 or 22 or 23 or 24 or 25 or 26 or 27 or 28 or 29 or 30 or 31 or 32 or 33 or 34 or 35 (82955)

37 19 and 36 (10248)

38 limit 37 to human (8482)

39 from 38 keep 1-1000 (1000)

***************************

Database: CINAHL - Cumulative Index to Nursing & Allied Health Literature <1982 to February Week 4 2007>

Search Strategy:

--------------------------------------------------------------------------------

1 opinion leader.mp. [mp=title, subject heading word, abstract, instrumentation] (18)

2 facilitat$.mp. [mp=title, subject heading word, abstract, instrumentation] (15597)

3 education outreach$.mp. [mp=title, subject heading word, abstract, instrumentation] (20)

4 academic detail$.mp. [mp=title, subject heading word, abstract, instrumentation] (46)

5 practice develop$.mp. [mp=title, subject heading word, abstract, instrumentation] (3397)

6 clinical educator.mp. [mp=title, subject heading word, abstract, instrumentation] (41)

7 exp Clinical Nurse Specialists/ or clinical nurse.mp. (6609)

8 change agen$.mp. [mp=title, subject heading word, abstract, instrumentation] (425)

9 knowledge broker$.mp. [mp=title, subject heading word, abstract, instrumentation] (4)

10 champion.mp. (288)

11 innovat$.mp. [mp=title, subject heading word, abstract, instrumentation] (9855)

12 boundary spann$.mp. [mp=title, subject heading word, abstract, instrumentation] (15)

13 advoca$.mp. [mp=title, subject heading word, abstract, instrumentation] (12804)

14 expert.mp. [mp=title, subject heading word, abstract, instrumentation] (6522)

15 exp EXPERT NURSES/ or exp EXPERT CLINICIANS/ or exp EXPERT SYSTEMS/ (1388)

16 exp Nursing Leaders/ or exp Leadership/ or transformational leader$.mp. (10192)

17 consultan$.mp. [mp=title, subject heading word, abstract, instrumentation] (5086)

18 coach$.mp. (1473)

19 educator.mp. or exp HEALTH EDUCATORS/ or exp DIABETES EDUCATORS/ or exp CHILDBIRTH EDUCATORS/ (3112)

20 knowledge trans$.mp. (657)

21 knowledge uptake.mp. (6)

22 exp Nursing Knowledge/ or exp Knowledge/ or knowledge utilization.mp. (12571)

23 knowledge exchange.mp. (31)

24 exp MEDICAL PRACTICE, RESEARCH-BASED/ or exp NURSING PRACTICE, RESEARCH-BASED/ or exp HEALTH SERVICES RESEARCH/ or exp PROFESSIONAL PRACTICE, RESEARCH-BASED/ or exp PHYSICAL THERAPY PRACTICE, RESEARCH-BASED/ (9511)

25 research uptake.mp. (58)

26 research utilization.mp. (1830)

27 research utilisation.mp. (48)

28 knowledge utilisation.mp. (1)

29 evidence based.mp. (14454)

30 evidence informed.mp. (16)

31 research trans$.mp. [mp=title, subject heading word, abstract, instrumentation] (4239)

32 organizational innovation.mp. [mp=title, subject heading word, abstract, instrumentation] (216)

33 exp "DIFFUSION OF INNOVATION"/ (1164)

34 1 or 2 or 3 or 4 or 5 or 6 or 7 or 8 or 9 or 10 or 11 or 12 or 13 or 14 or 15 or 16 or 17 or 18 or 19 (69431)

35 20 or 21 or 22 or 23 or 24 or 25 or 26 or 27 or 28 or 29 or 30 or 31 or 32 or 33 (40961)

36 34 and 35 (5638)

37 from 36 keep 1-1000 (1000)

Database: PsycINFO

Search Strategy:

--------------------------------------------------------------------------------

1 opinion leader.mp. (24)

2 facilitat$.mp. [mp=title, abstract, heading word, table of contents, key concepts] (41811)

3 education outreach$.mp. [mp=title, abstract, heading word, table of contents, key concepts] (16)

4 academic detail$.mp. [mp=title, abstract, heading word, table of contents, key concepts] (32)

5 practice develop$.mp. (209)

6 clinical educator.mp. (10)

7 change agen$.mp. [mp=title, abstract, heading word, table of contents, key concepts] (618)

8 knowledge broker$.mp. (11)

9 champion.mp. (226)

10 innovat$.mp. [mp=title, abstract, heading word, table of contents, key concepts] (12735)

11 innovat$.mp. [mp=title, abstract, heading word, table of contents, key concepts] (12735)

12 boundary spann$.mp. [mp=title, abstract, heading word, table of contents, key concepts] (150)

13 advoca$.mp. [mp=title, abstract, heading word, table of contents, key concepts] (12409)

14 expert.mp. (11444)

15 exp Transformational Leadership/ or transformational leader$.mp. (793)

16 consultan$.mp. [mp=title, abstract, heading word, table of contents, key concepts] (4989)

17 coach$.mp. [mp=title, abstract, heading word, table of contents, key concepts] (3579)

18 educator.mp. (1538)

19 1 or 2 or 3 or 4 or 5 or 6 or 7 or 8 or 9 or 10 or 11 or 12 or 13 or 14 or 15 or 16 or 17 or 18 (86102)

20 knowledge trans$.mp. (427)

21 knowledge uptake.mp. (4)

22 knowledge utilization.mp. (55)

23 knowledge utilisation.mp. (0)

24 research uptake.mp. (2)

25 exp Evidence Based Practice/ or evidence based$.mp. (5371)

26 evidence informed$.mp. (23)

27 innovation.mp. (4051)

28 research use$.mp. (706)

29 research trans$.mp. (56)

30 organizational innovation.mp. (104)

31 organisational innovation.mp. (2)

32 diffusion of innovation.mp. (74)

33 20 or 21 or 22 or 24 or 25 or 26 or 27 or 28 or 29 or 30 or 31 or 32 (10556)

34 19 and 33 (4993)

35 from 34 keep 1-1000 (1000)

***************************

Search Strategy

Web of Science

March 9, 2007

(TI=knowledge trans* OR TI=knowledge utili?ation OR TI=knowledge exchange OR TI=knowledge uptake OR TI=knowledge use OR TI=research trans* OR TI=research utili?ation OR TI=research uptake OR TI=research use OR TI=evidence based OR TI=evidence informed OR TI=innovation OR TI=organi?ational innovation OR TI=diffusion of innovation) AND (TI=opinion leader OR TI=facilitator OR TI=facilitation OR TI=education outreach work* OR TI=academic detail* OR TI=practice develop* OR TI=clinical educator OR TI=change agen* OR TI=knowledge broker OR TI=knowledge brokers OR TI=knowledge brokering OR TI=champion OR TI=innovator OR TI=boundary spann* OR TI=advoca* OR TI=expert OR TI=transformational leader OR TI=transformational leader* OR TI=consultant OR TI=consultan* OR TI=coach OR TI=educator)

CSA

Multiple Databases

Query: (((DE=("information dissemination" or "diffusion" or "knowledge

utilization")) and (DE="evidence based practice") and (DE=("diffusion" or

"adoption of innovations")) and (DE=("innovations" or "technological

innovations"))) or (((knowledge trans*) or (knowledge uptake) or

(knowledge use)) or ((knowledge utili?ation) or (knowledge exchange) or

(research trans*)) or ((research uptake) or (research use) or (research

utili?ation)) or ((evidence base*) or (evidence informed) or innovation)

or ((organi?ational innovation) or (diffusion of innovation)))) and

(((opinion leaders) or (opinion leader) or facilitat*) or ((education

outreach worker) or (academic detail*) or (practice develop*)) or

((clinical educator) or (change agents) or (change agent)) or ((change

agency) or (knowledge brok*) or champion) or (innovator or (boundary

spann*) or advocacy) or (advocate or experts or expert) or

((transformational lead*) or consultants or consultant))

Your Comments: soc abstracts results
