# Supplementary material for: Realist synthesis: illustrating the method for implementation research
Source: Implement Sci. 2012 Apr 19;7:33. doi: 10.1186/1748-5908-7-33 (PMC3514310; doi:10.1186/1748-5908-7-33)
Supplement: Additional file 5 — Stages of synthesis. [file 1748-5908-7-33-S5.doc]

Step 1: Organising extracted data from both reviewers into evidence tables

| **THEORY AREA 1 - What impact do the characteristics of the change agent have on KU?** | | **COMMENTS** |
| --- | --- | --- |
| **Moore, KA; Peters, RH; Hills, HA; Levasseur, JB;**  **Rich, AR; Hunt, WM; Young, MS, Valente, TW (2004)** | The study identified a number of ‘competency – related’ characteristics of opinion leaders (OL) that are significant, including – postgraduate education; relevant professional credentials; years of experience in the treatment area. Identified opinion leaders (n=10) were compared with other counsellors (n=55).  In this study, OLs were found to have more postgraduate education, more relevant professional credentials and more postgraduate qualifications.  “OLs had significantly more work experience in mental health than their co-workers, and were twice as likely to have postgraduate education in comparison to their peers … OLs were more confident and willing to work with clients who have co-occurring disorders. In comparison to their colleagues, OLs had significantly greater knowledge regarding diagnosis and treatment of co-occurring disorders” (p199) |  |
| **Stanley D (2006)** | This paper does not specifically focus on KU but it is still helpful to KU with its focus on the characteristics of clinical leaders.  A clinical leader is defined as “one who possesses clinical expertise in a specialty practice area and who uses interpersonal skills to enable nurses and other healthcare providers to deliver quality patient care” (p108).  The study of the characteristic of clinical leaders suggests “… it is the demonstration and translation of their values and beliefs into the actions and the functions of their role for which they are admired and followed” (p110). |  |

Step 2: Themes from each article are identified by two reviewers

Reviewer 1

| **Moore, KA; Peters, RH; Hills, HA; Levasseur, JB; Rich, AR; Hunt, WM; Young, MS, Valente, TW (2004)** | The study identified a number of ‘competency – related’ characteristics of opinion leaders (OL) that are significant, including – postgraduate education; relevant professional credentials; years of experience in the treatment area. Identified opinion leaders (n=10) were compared with other counsellors (n=55).  In this study, OLs were found to have more postgraduate education, more relevant professional credentials and more postgraduate qualifications.  “OLs had significantly more work experience in mental health than their co-workers, and were twice as likely to have postgraduate education in comparison to their peers … OLs were more confident and willing to work with clients who have co-occurring disorders. In comparison to their colleagues, OLs had significantly greater knowledge regarding diagnosis and treatment of co-occurring disorders” (p199) | **More Postgraduate education, more postgraduate qualifications and more professional credentials were found among opinion leaders than other counsellors.** |
| --- | --- | --- |

Reviewer 2

| **Moore, KA; Peters, RH; Hills, HA; Levasseur, JB; Rich, AR; Hunt, WM; Young, MS, Valente, TW (2004)** | The study identified a number of ‘competency – related’ characteristics of opinion leaders (OL) that are significant, including – postgraduate education; relevant professional credentials; years of experience in the treatment area. Identified opinion leaders (n=10) were compared with other counsellors (n=55).  In this study, OLs were found to have more postgraduate education, more relevant professional credentials and more postgraduate qualifications.  “OLs had significantly more work experience in mental health than their co-workers, and were twice as likely to have postgraduate education in comparison to their peers … OLs were more confident and willing to work with clients who have co-occurring disorders. In comparison to their colleagues, OLs had significantly greater knowledge regarding diagnosis and treatment of co-occurring disorders” (p199) | **Characteristics influencing practice knowledge/competence were:**  **Post graduate education**  **Prof. credentials**  **Yrs of experience**  **Confidence**  **Willing to work with clients w/ co-disorders** |
| --- | --- | --- |

Step 3: Themes identified by different reviewers and amalgamated and chains of inference are established

| **Author** | **Individual comments** | **Step 2. Amalgamated themes** | **Step 3: Look for chain of inferences (connections across papers) to ultimately develop hypotheses** | **Decision/recommendations of conference call** |
| --- | --- | --- | --- | --- |
| 1) Moore, KA; Peters, RH; Hills, HA; Levasseur, JB; Rich, AR; Hunt, WM; Young, MS, Valente, TW (2004) | Does not address impact change agent characteristics on KU  but does address characteristics of OL in practice/competence | 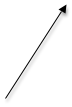Post graduate education  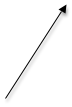Prof. credentials  Yrs of experience  Confidence  Greater knowledge | Expert Knowledge  Professional qualifications  Experience  Positive attitude | Need to go back to article and determine if “confidence” is related to knowledge or experience;  Possibly remove positive attitude based on review of article |

Step 4: Articles containing the themes used to derive the chains of inference are identified

| **Chains of Influence** | **Derived from the following themes in step 3** | **Articles** |
| --- | --- | --- |
| Knowledge | Professional qualifications  Expert knowledge  Knowledgeable  Local knowledge  Research Knowledge  Practice knowledge | 1,3, 6,7,10, 11, 13, 14,15, 16,18,19,20,21, 22, 23, 25, 29,35,36,37,39 |
| Skills | Communication skills  Leadership skills  Thinking skills  Clinical skills  Cognitive skills  Evaluation skills  Political skills  Facilitation Skills  Reflective skills | 2, 4,5,6,7,8,9,10,11,12, 13, 14,15,16, 17, 18,19,20,21, 22, 24,25,27,28,32, 33, 34,36,38,39,40 |
| Personal Characteristics | Role model  Positive attitude  Responsibility/accountability  Respected  Information Seeking  Positive Attitude  Accessible  Age  Teacher  Culturally compatible  Objectivity  Years of experience | 1,2,4, 6,7,8, 13, 14, 15, 16, 17,18, 22,28,29,30,31,32,33,35,36, 37,38, 39 |
| Social Interaction | Social Influence  Networking  Shared Ownership | 5,8,12, 15,18,31,39,40, Wright |

Step 5: Making connections among chains of inference

| **Nature of relationship …**  between CA personal characteristics and the ability to form partnerships on the impact of EIHC.  among CA personal characteristics, role adopted, and contextual influences on the impact of EIHC.  between CA personal characteristics, skills, and knowledge and the interplay with contextual influences on the impact of EIHC.  between knowledge and human personal sources of information on the impact of EIHC.  among contextual influence, social interaction, CA personal characteristics and human personal sources of information and impact on the EIHC.  among partnership, social interaction, contextual influence, and skills on the impact of EIHC.  between skills and roles on the impact of EIHC. |
| --- |

Step 6: Generation of hypotheses using the chains of inference

| Hypotheses | Chain of Inference (theory level) | Chain of inference (sub-theory level) | Themes from the literature | Papers addressing the theme |
| --- | --- | --- | --- | --- |
| An opinion leader and his/her personal characteristics are dependent on contextual factors in order to have an impact on EIHC.  A facilitator and his/her personal characteristics are dependent on contextual factors in order to have an impact on EIHC. | The nature of the relationship between the change agent’s personal characteristics, the role adopted, and contextual influences and the impact of EIHC. | Roles  Personal Characteristics  Contextual Factors | Opinion Leader  Facilitator | *Papers with mixed and positive effects, only:*  6 OL (Wright, Chaillet, Curran, Moore, Davies, Majumdar)    6 FAC (int/ext and ext fac incl), (Stetler, Cranney, Gerrish, Milner, Thomas, Hutt)  Total 18 CA papers, 12 OL and FAC |
