# Supplementary material for: Realist synthesis: illustrating the method for implementation research
Source: Implement Sci. 2012 Apr 19;7:33. doi: 10.1186/1748-5908-7-33 (PMC3514310; doi:10.1186/1748-5908-7-33)
Supplement: Additional file 6 — Hypotheses linked to themes, chains of inference and papers. [file 1748-5908-7-33-S6.doc]

**HYPOTHESES GENERATED**

| **Hypothesis 1** | **Chain of inference (theory level)** | | **Chain of inference (sub-theory level)** | | **Themes from literature** | | **Papers addressing theme** | ***Papers with outcomes data*** |
| --- | --- | --- | --- | --- | --- | --- | --- | --- |
| A change agent and his/her personal characteristics are dependent on contextual factors in order to have an impact on EIHC. | 1 and 3 | | Change agent roles in general | | Opinion Leader  Facilitator (external facilitator and int/ext facilitator)  Not used in these hypotheses but are in this sub-theory level:  Practice Developer  Academic Detailer  Educational Outreach  Multiple Change Agents | | *Papers with mixed and positive effects, only:*  6 OL (Wright, Chaillet, Curran, Moore, Davies, Majumdar)    6 FAC (int/ext and ext fac incl), (Stetler, Cranney, Gerrish, Milner, Thomas, Hutt)  Not used in these hypotheses but are in this sub-theory level:  3 PD (Wright et al 2001, Garbett 2006, Wright & McCormack)  2 Educ Outreach (Seager, Bernal-Delgado)  1 AD (Funk)  1 multiple CA (Reymond)  Total 18 papers | Milner meas’d impact,  Arsanow possible dose  Wright dose  Majumdar good quantification  Bernal-Delgado  Goldman ES but what is the CA?  Funk (need to check paper for ES) |
| **Roles** | | **Personal Characteristics** | | **Contextual Influences** | | **Evaluation of the lit** | | |
| 1 – opinion leaders  1 – facilitators  2 – practice developer  3 – educational outreach  4 – academic detailing  5 – multiple CA  (18 papers) | | -accessible  -respected  -age (youth)  -positive attitudes  -responsibility/accountability  - cultural compatibility  -reflective  -role model  (5 papers) | | -leadership  -embeddedness  -culture  -environment  (14 papers) | | -literature predominantly focuses on the overall impact of roles but not characteristics  -while authors may describe personal characteristics, there is little work to measure the impact  -facilitator vs. facilitation (not clear which is which, terms may have been used interchangeably)  -sloppy use of language in general; important in the field to be clear with use of terms | | |

| **Hypothesis 2** | **Chain of inference (theory level)** | | **Chain of inference (sub-theory level)** | | **Themes from literature** | | **Papers addressing theme** | ***Papers with outcomes data*** |
| --- | --- | --- | --- | --- | --- | --- | --- | --- |
| The role and personal characteristics of a change agent impacts on the degree and type of influence that he/she has on context | 2 | | Specific change agent roles and their potential impact | |  | |  |  |
| **Roles** | | **Personal Characteristics** | | **Contextual Influences** | | **Evaluation of the lit** | | |
| 1 – opinion leaders  1 – facilitators  2 – practice developer  3 – educational outreach  4 – academic detailing  5 – multiple CA  (18 papers) | | -accessible  -respected  -age (youth)  -positive attitudes  -responsibility/accountability  - cultural compatibility  -reflective  -role model  (5 papers) | | -leadership  -embeddedness  -culture  -environment  (14 papers) | | -literature predominantly focuses on the overall impact of roles but not characteristics  -while authors may describe personal characteristics, there is little work to measure the impact  -facilitator vs. facilitation (not clear which is which, terms may have been used interchangeably)  -sloppy use of language in general; important in the field to be clear with use of terms | | |

| **Hypothesis 3** | **Chain of inference (theory level)** | | **Chain of inference (sub-theory level)** | | **Themes from literature** | | **Papers addressing theme** | ***Papers with outcomes data*** |
| --- | --- | --- | --- | --- | --- | --- | --- | --- |
| The interplay of change agents’ skills and knowledge with contextual factors impacts on EIHC | 3, 4, 5 and 7 | |  | |  | |  |  |
| **Roles** | | **Personal Characteristics** | | **Contextual Influences** | | **Evaluation of the lit** | | |
| 1 – opinion leaders  1 – facilitators  2 – practice developer  3 – educational outreach  4 – academic detailing  5 – multiple CA  (18 papers) | | -accessible  -respected  -age (youth)  -positive attitudes  -responsibility/accountability  - cultural compatibility  -reflective  -role model  (5 papers) | | -leadership  -embeddedness  -culture  -environment  (14 papers) | | -literature predominantly focuses on the overall impact of roles but not characteristics  -while authors may describe personal characteristics, there is little work to measure the impact  -facilitator vs. facilitation (not clear which is which, terms may have been used interchangeably)  -sloppy use of language in general; important in the field to be clear with use of terms | | |

| **Hypothesis 4** | **Chain of inference (theory level)** | | **Chain of inference (sub-theory level)** | | **Themes from literature** | | **Papers addressing theme** | ***Papers with outcomes data*** |
| --- | --- | --- | --- | --- | --- | --- | --- | --- |
| The social interaction of a change agent and his/her ability to work in partnership impacts on EIHC | 1, 5 and 6 | |  | |  | |  |  |
| **Roles** | | **Personal Characteristics** | | **Contextual Influences** | | **Evaluation of the lit** | | |
| 1 – opinion leaders  1 – facilitators  2 – practice developer  3 – educational outreach  4 – academic detailing  5 – multiple CA  (18 papers) | | -accessible  -respected  -age (youth)  -positive attitudes  -responsibility/accountability  - cultural compatibility  -reflective  -role model  (5 papers) | | -leadership  -embeddedness  -culture  -environment  (14 papers) | | -literature predominantly focuses on the overall impact of roles but not characteristics  -while authors may describe personal characteristics, there is little work to measure the impact  -facilitator vs. facilitation (not clear which is which, terms may have been used interchangeably)  -sloppy use of language in general; important in the field to be clear with use of terms | | |
